# Epidemiology of imported malaria among children and young adults in Barcelona (1990-2008)

**DOI:** 10.1186/1475-2875-10-347

**Published:** 2011-11-25

**Authors:** Mireia Garcia-Villarrubia, Juan-Pablo Millet, Patricia Garcia de Olalla, Joaquim Gascón, Victoria Fumadó, Jordi Gómez i Prat, Begoña Treviño, María-Jesús Pinazo, Juan Cabezos, José Muñoz, Francesc Zarzuela, Joan A Caylà

**Affiliations:** 1 Epidemiology Service, Public Health Agency of Barcelona, Pza Lesseps, 1, 08023 Barcelona, Spain; 2 CIBER de Epidemiología y Salud Publica (CIBERESP), Spain; 3 Hospital Clínic, Barcelona Centre for International Health Research (CRESIB), Hospital Clínic/IDIBAPS, Barcelona, Spain. Villarroel 170, 08036 Barcelona, Spain; 4 Sant Joan de Deu Paediatrics Hospital. Passeig Sant Joan de Déu, 2. 08950 Esplugues de Llobregat, Barcelona, Spain; 5 Tropical Medicine and International Health Unit, Primary Health Care Drassanes Center, Avda Drassanes 17-21, 08001 Barcelona, Spain

## Abstract

**Background:**

Increasing international travel and migration is producing changes in trends in infectious diseases, especially in children from many European cities. The objective of this study was to describe the epidemiology and determine the trends of imported malaria in patients under 20 years old in the city of Barcelona, Spain, during an 18-year period.

**Methods:**

The study included malaria cases that were laboratory confirmed and reported to the malaria register at the Public Health Agency of Barcelona from 1990 to 2008, residing in Barcelona and less than 20 years old. Patients were classified as natives (born in Spain) or immigrants. Differences in the distribution of demographic, clinical characteristics, and incidence per 100,000 person-year evolution were analysed. Natives and immigrants were compared by logistic regression by calculating the *odds ratio *(OR) with a 95% confidence interval (CI) and Chi-square for a linear trend (p < 0.05).

**Results:**

Of the total 174 cases, 143 (82.1%) were immigrants, 100 (57.5%) were female, 121 (69.5%) *Plasmodium falciparum*, and 108 (62.1%) were visiting friends and relatives (VFR) as the reason for travel. Among the immigrants, 99 (67.8%) were from Equatorial Guinea. Immigrant cases more frequently travelled to Africa than natives (p = 0.02). The factors associated with imported malaria among immigrant residents was travelling for VFR (OR: 6.2 CI 1.9-20.2) and age 15-19 (OR: 3.7 CI 1-13.3). The incidence increased from 1990 to 1999 (p < 0.001) and decreased from 2000 to 2008 (p = 0.01), although the global linear trend was not statistically significant (p = 0.41). The fatality rate was 0.5%.

**Conclusions:**

The majority of cases of malaria in population less than 20 years in Barcelona were immigrants, travelling to Africa for VFR and *Plasmodium falciparum *was most frequently detected. The trend analysis of the entire study period did not show a statistically significant decline. It is recommended to be aware of malaria, especially among children of immigrants who travel to their parent's home country for VFR. Better access to pre travel advice should be provided.

## Background

The study of imported malaria has become of more interest in recent years than ever in before. The concern within the scientific community reflects a problem that is secondary to increasing international travel to endemic regions and recent immigration from low-income countries [1,2]. Identify and define this problem in non-endemic countries, has allowed to identify vulnerable populations in various settings [3-16]. Malaria is one of the most important infectious diseases that affect children after an international travel [13]. Infection by *Plasmodium falciparum *and immigration from sub-Saharan Africa are characteristics of the majority of imported malaria cases in Spain [7-9], as well as in other European countries, and of the United States (EUA) [13-17].

Imported malaria characteristics and incidence evolution for vulnerable populations such as children are still not well understood in spite of a disproportionally high number of cases [7,11]. For example, 15-20% of the total reported cases of imported malaria correspond to children and young adults [7,12,18]. This population deserves special attention because it may be producing a significant increase in morbidity and mortality of a preventable disease [13].

Affected children in European countries and cities are typically resident immigrants of non-endemic countries who travel to their country of birth or their parent's birthplace to visit friends and relatives (VFR) [13,19-21]. Recent studies have also shown poor adherence to chemoprophylaxis (CP) and a higher probability to be hospitalized among this population [7,9,13,20,22].

Population-based studies, which would show incidence rate evolution and define the problem, are scarce. The objective of this study was to describe the epidemiological characteristics of imported malaria cases among children and young adults under 20 years of age and to examine the incidence evolution in a European city during a long period of time. Identification of the patient profile will allow for a connection between specialized healthcare centres and public health measures to prevent imported malaria in children and young adults.

## Methods

### Population and study period

A population-based observational study design was implemented to examine confirmed imported malaria cases in children less than 20 years of age, residents of the city of Barcelona, and reported to the malaria registry of the Public Health Agency of Barcelona (Spain) Epidemiology Service between January 1, 1990 and December 31, 2008. Relapses (cases with new symptoms and/or parasitaemia who have not travelled again) were excluded. Reporting imported malaria cases to public health is mandatory in Spain. In Barcelona a laboratory surveillance system exists but cases are reported by the clinicians. Epidemiological surveys are completed by public health nurses.

### Definitions

*Imported malaria *was defined as acquired in an endemic area and compliant with diagnostic criteria of microscopic observation of parasites in a thick peripheral blood smear or genomic detection by amplification techniques in a non-endemic country [23]. An *immigrant *was defined as born in an endemic area and resident of the city of Barcelona. *VFR immigrant *was defined as having travelled to their country of origin to visit friends and relatives, and a *recently arrived immigrant *was defined as having arrived in Barcelona with malaria before residency. Immigrant children born in Barcelona were considered *natives *[24].

### Laboratory

Cases and species were confirmed by microscopic observation of parasites in a thick and thin blood film and by PCR as previously described [7].

### Variables

The following variables from the epidemiology survey for each case were systematically reviewed: socio-demographic characteristics (sex, age, country of birth, place of residence), epidemiological characteristics (reporting healthcare centre, hospitalization, endemic geographical region visited in the 30 days previous to symptom onset), reason for travel (VFR, work, volunteer or recently arrived), diagnostic variables (species and technique), chemoprophylaxis, treatment, and date of symptom onset, diagnosis, hospitalization admittance and discharge. Laboratory registries and clinical histories were reviewed at the tropical disease reference centre of Barcelona for additional information.

### Statistical analysis

A descriptive analysis of the variables was performed. Quantitative variables were described using medians and interquartile ranges (IR) according to their no normal distribution. The x^2 ^test was used to compare qualitative variables. ANOVA and corresponding non-parametric tests were used to compare quantitative variables. Incidence rates were calculated per 100,000 person-years according to the total population average for a five-year period (data was obtained from the city census) [24]. Recently arrived immigrants were excluded from incidence rate calculations. Incidence evolution during the study period was analysed using an x^2 ^and adjusted linear trend test.

Differences between immigrant and native cases on bivariate and multivariate levels were compared using a logistic regression model and *odds ratio *(OR) with a 95% confidence interval (CI). Variables from the bivariate model with a p-value less than 0.05 and those of epidemiological interest were included in the multivariate model. Fatality rates were calculated according to the total number of deaths and total cases per 100. Statistical package SPSS 18 and Epi Info 6 were used for the statistical analysis.

## Results

The 174 cases reported during the study period were included. The median age was 9.6 years (IR: 5-15 years), one hundred cases (57.5%) were female and 143 (82.1%) were immigrants. The majority of cases had travelled to Africa (146 cases, 83.9%), of which 53.4% travelled to Equatorial Guinea (EG) and 8% to Cameron. Of the 125 cases who were prescribed chemoprophylaxis, almost all did not take it correctly (121 cases, 96.8%). The most common reason for travel was VFR (108 cases, 62.1%) and the most commonly detected species was *P. falciparum *(121 cases, 69.5%).

**Figure 1:**
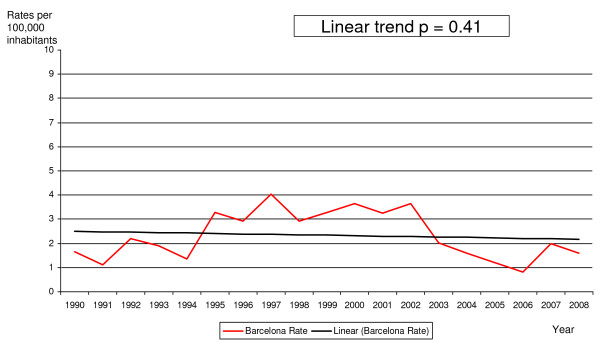
**Evolution of imported malaria rates in cases under 20 years of age**. Barcelona 1990-2008.

Imported malaria incidence increased from 1.65 cases per 100,000 persons under 20 years old in 1990 to four cases per 100,000 in 1997, at which point it declined to reach 1.6 cases per 100,000 in 2008. The linear trend analysis showed a significant increase from 1990 to 2000 (p < 0.001) and a decrease from 2001 to 2008 (p = 0.01). However, analysis of the entire study period did not show a statistically significant decline (p = 0.41) (Figure 1).

Table 1 summarizes the epidemiological characteristics of native and immigrant cases.

**Table 1:** Characteristics of 174 reported malaria cases in immigrants and natives under 20 years of age in Barcelona, 1990-2008.

	Immigrant n(%)	Native n (%)	Total n (%)	p-value
**Sex**				
Male	60 (42.0)	14 (45.2)	74 (42.5)	0.56
Female	83 (58.0)	17 (54.8)	100 (57.5)	

**Age (years)**				
< 5	40 (28.0)	13 (41.9)	53 (30.5)	0.65
6 to 14	59 (41.3)	12 (38.7)	71 (40.8)	0.24
15 to 19	44 (30.8)	6 (19.4)	50 (28.7)	

**District of residence**				
Other	119 (83.2)	27 (87.1)	146 (83.9)	0.19
Inner City	24 (16.8)	4 (12.9)	28 (16.1)	

**Continent of travel**				
Africa	125 (87.4)	21 (67.7)	146 (83.9)	0.02
Asia	11 (7.7)	4 (12.9)	15 (8.6)	
America	3 (2.1)	-	3 (1.7)	
Unknown	4 (2.8)	6 (19.4)	10 (5.7)	

**Reason for travel (VFR)**				
No	55 (38.5)	11 (35.5)	66 (37.9)	0.88
Yes	88 (61.5)	20 (64.5)	108 (62.1)	

**Species detected**				
*P. falciparum*	103 (72)	18 (58.1)	121 (69.5)	0.64
*P. vivax*	18 (12.6)	7 (22.6)	25 (14.4)	
*P. ovale*	7 (4.9)	3 (9.7)	10 (5.7)	
*P. malariae*	7 (4.9)	1 (3.2)	8 (4.6)	
*Mixed*	4 (2.8)	1 (3.2)	5 (2.9)	
*Plasmodium spp*	4 (2.8)	1 (3.2)	5 (2.9)	

**Chemoprophylaxis**				
Yes	3 (2.1)	1 (3.2)	4 (2.3)	0.53
No	140 (97.9)	30 (96.8)	170 (97.7)	

**Hospitalization**				
Yes	22 (15.4)	5 (16.1)	27 (15.5)	0.7
No	121(84.6)	26 (83.9)	147 (84.5)	

VFR: *visiting friends and relatives*.

Immigrant cases more frequently visited Africa compared to their native counterparts (0.02). Excluding recently arriving immigrants with malaria, no difference in sex, district of residence, country of travel or detected species between resident immigrants and natives were found. Similarly, no difference was seen in hospitalization or chemoprophylaxis completion between the two groups (Table 2). The factors associated with imported malaria among young resident immigrants in Barcelona were age between 15 and 19 years (OR: 3.7, CI 1-13.3) and VFR (OR 6.4, CI 2-19.9).

**Table 2:** Characteristics of malaria in 96 resident immigrants and 29 native cases under 20 years of age in Barcelona 1990-2008*.

	Resident immigrant n(%)	Native n (%)	Total n (%)	Crude OR (95%CI)	Adjusted OR (95%CI)	P value
**Sex**						
Female	54 (56.3)	14 (48.3)	68 (54.4)	1	1	0.41
Male	42 (43.8)	15 (51.7)	57 (45.6)	0.7 (0.3-1.7)	0.7 (0.3-1.7)	

**Age (years)**						
< 5	27 (27.8)	12 (42.9)	39 (31.2)	1	1	0.044
6 to 14	41 (42.3)	11 (39.3)	52 (41.6)	1,4 (0.6-3.8)	1.9 (0.7-5.3)	
15 to 19	29 (29.9)	5 (17.8)	34 (27.2)	2,6 (0.8-8.3)	3.7 (1-13.3)	

**District of residence**						
Other	79 (81.4)	24 (85.7)	103 (82.4)	1		
Inner city	18 (18.6)	4 (14.3)	22 (17.6)	1.4 (0.5-4.7)		

**Continent of travel**						
Asia	9 (9.4)	4 (16.7)	13 (10.8)	1		
America	3 (3.1)	0 (0)	3 (2.5)	1		
Africa	84 (87.5)	20 (83.3)	104 (86.3)	2,4 (0.9-6.6)		

**Reason of travel (VFR)**						
No	9 (9.3)	8 (28.6)	17 (13.6)	1	1	0.003
Yes	88 (90.7)	20 (71.4)	108 (86.4)	5 (1.7-14.4)	6.4 (2-19.9)	

**Species detected**						
*P. falciparum*	68 (70.1)	16 (57.1)	84 (67.2)	1.6 (0.7-3.9)		
*P. vivax*	14 (14.4)	6 (21.4)	20 (16)	1		
*P. ovale*	4 (4.1)	3 (10.7)	7 (5.6)			
*P. malariae*	3 (3.1)	1 (3.6)	4 (3.2)			
*Mixed*	4 (4.1)	1 (3.6)	5 (4)			
*Plasmodium spp*	4 (4.1)	1 (3.6)	5 (4)			

**Chemoprophylaxis**						
No	93 (69.9)	28 (96.6)	121 (96.8)	1		
Yes	3 (3.1)	1 (3.4)	14 (3.2)	0.9 (0.09-9)		

**Hospitalization**						
No	87 (90.6)	24 (82.8)	111 (88.8)	1		
Yes	9 (9.4)	5 (17.2)	14 (11.2)	0.5 (0.2-1.6)		

* 49 recently arrived immigrants were excluded.

Among the 108 cases with VFR as the reason for travel, 31 cases (28.7%) had a previous episode of malaria in their life, 97.2% did not correctly complete chemoprophylaxis and 27 cases (11.1%) required hospitalization. Six cases (6.5%) were hospitalized between 1990 and 1999, and 21 cases (26.3%) were hospitalized between 2000 and 2008 (p < 0.001). During the 18-year study period, one death due to malaria was reported (fatality rate of 0.54%).

## Discussion

Imported malaria in young adults of Barcelona predominantly affected immigrants over five years of age who travelled to Africa (most frequently EG) to visit friends and relatives, did not complete chemoprophylaxis, were infected with *P. falciparum *and were treated without need of hospitalization. Age 15-19 years and VFR were associated factors to resident immigrants compared to natives. This study also found a decline in imported malaria incidence in recent years, which was not statistically significant.

As described in adult populations of similar settings in Spain and other European countries [8,10,13,15,16,22-25], the majority of children affected by imported malaria in this study were African immigrants or children of African immigrants who travelled to sub-Saharan Africa [9,13,26,27]. A recent analysis from the GeoSentinel Surveillance Network found that 69% of children needed hospitalization. *Plasmodium falciparum *accounted for 78% of all malaria cases, 95% of which were acquired in sub-Saharan Africa [13]. A high number of malaria cases were found in EG immigrants and their children, which has been analysed in previous studies performed in Barcelona and other Spanish cities [18,25,28]. This high number of cases among Guinean population and the existence of two Tropical Medicine Units in the city, could explain the low percent of hospitalizations in Barcelona.

Many immigrants that are established residents in Spain visit their home country [29] with their children, who have rarely or never been exposed to malaria and thus possess little or null natural semi-immunity. These travellers are not accustomed to seek pre-travel advice or take chemoprophylaxis, although they travel to rural areas for longer periods of time [4,7,19]. A big concern is what Hagmann *et al *described in a recent study; VFR children were less likely than adults to receive pre travel medical advice [13]. Like the rest of the travellers, resident immigrants and children who travel should be educated about the various preventative measures, such as barriers, impregnated nets and chemoprophylaxis completion for travel to endemic regions. Innovative methods to improve access to pre-travel services for VFR should be implemented [13,14].

The association between imported malaria and VFR in patients under 20 years old has been reported in various European cities and countries [13,18,30] and was thoroughly described in a recent study about childhood malaria in England and Ireland [12]. The characteristics of imported malaria cases found in our study, such as VFR travel reason, *P. falciparum *infection, and incomplete chemoprophylaxis use, are similar to those described in other cosmopolitan cities of the world [3,21,22]. *Plasmodium falciparum *is the most frequently identified species in children [26,30,31] not only in Barcelona, but also in the rest of Spain [8,11,32-36], other European countries [13,15,17,26] and in the USA [6,37,38].

The infrequent use of chemoprophylaxis is a concern on a global scale. Although anti-malarial drug resistances are emerging in endemic countries, chemoprophylaxis is still effective and selection of a good regimen, along with barrier and repellent precautions, is key to decreasing the risk of acquiring malaria [39]. The rate of correct chemoprophylaxis completion among cases in this study population is similar to that found in previous studies, which ranges from 3-15% [19,31,38]. These results demonstrate the severity of this problem. However, further evaluation is needed as we do not know how many people in the same age group in the general population travelled and performed chemoprophylaxis correctly. One explanation about the low chemoprophylaxis completion could be the false cultural idea that people born in endemic regions and their families are protected against malaria [22], underestimating the importance of disease and its potential fatality. It is also important to note that 28.7% of the resident immigrants travelling for VFR who did not complete chemoprophylaxis had previously suffered from malaria. Nonetheless, it would be important in the future differentiate if the previous malaria episode was in all the patients life or as a VFR. However, this percent is similar to the 26% found in a study from England in 2007 [12], and exposes an important missed opportunity for patient education about disease prevention. For example, assuming 90% effectiveness and 90% adherence, 105 of the 129 cases of malaria in resident immigrants could have been avoided.

The increase in incidence from 1990 to 1999 can be attributed to the high in international travel to endemic regions and infrequent use of chemoprophylaxis. The later decrease in incidence between 2000 and 2008 could be due to decreased incidence in endemic countries by the use of artemisinins and mosquito control programmes. Better travel advice, higher quality of travel health information in hospitals and primary care facilities and the correct use of chemoprophylaxis could also had a role in the decrease of incidence. There are many other factors that could also affect the incidence calculations such as migration flow, the census development and case reporting. Nonetheless, according to the linear trend analysis, the decline over the entire study period was not significant. Malaria incidence among children had not been previously described but one study from The Netherlands reported decreasing incidence among adults [10]. The incidence of two cases per 100,000 reported in this study is slightly inferior to that reported in 2007 in the UK (2.8/100,000 per year) and Ireland (4.6/100,000 per year) in children less than 16 years of age [12]. This difference could be due the high amount of immigration from endemic countries to the UK in comparison to Catalonia or Spain. Although arguably low, this incidence is not satisfactory for a preventable disease.

Studies that have assessed imported malaria among children have showed the important aspects of the clinical epidemiological patterns in various non-endemic countries [19,40]. One strength of this study is the large study population. It is the first population-based study performed in Spain and provides incidence evolution over the last 18 years. The limitations of the study are the lack of clinical data information, reasons for hospitalization, as well as data about parents' country of birth. Immunology studies and patterns of travel among immigrants and the time spent as residents of a non-endemic country would also help us associate the disease with a level of semi-immunity in the future [41,42].

The fatality rate was similar to previous studies performed in industrialized cities, except in Italy, Japan, Great Britain and Sweden, in which no deaths were reported [12,26]. The vulnerability of the patients must not be forgotten, even though the lethality rate represents only one death. This patient was the son of immigrant parents from an endemic region, less than five years of age, who had travelled for VFR to a region of sub-Saharan Africa. He had never had malaria and did not complete chemoprophylaxis. Diagnostic delays of malaria are associated with higher rates of hospitalization and mortality, thus malaria should be considered for any child with a history of recent travel to an endemic country. This is especially important because malaria symptoms are non-specific and a diagnostic delay can be fatal [12,13,43].

Challenges for health systems in developed countries in the control of imported malaria include the improvement of information dissemination of preventative measures, the correct use of chemoprophylaxis when necessary and rapid diagnosis of clinical cases [43-45]. To reduce the risk of a diagnostic delay, protocols for primary care, emergency care and paediatric facilities should specify malaria as a possibility for immigrant patients and those who travel to an endemic region. Primary care teams working in areas of high immigration should also implement community activities to improve information availability and awareness. Furthermore, to promote their use, chemoprophylaxis recommendations should be available in most languages. Medicine is a dynamic and bio-psycho-social science in which many groups are involved and it is, therefore, necessary to quickly adapt to the needs of the population at any moment.

## Conclusions

The majority of cases of malaria in population less than 20 years in Barcelona were immigrants, travelling to Africa to VFR and *P. falciparum *was the most frequently detected species. It is recommended to be aware of infectious diseases, such as malaria, especially in children and young adults who travel to their parent's home country to visit friends and relatives. Furthermore, worldwide prevention programmes targeting these vulnerable populations should be enhanced by providing better access to travel advice to prevent the disease.

## Competing interests

The authors declare that they have no competing interests.

## Authors' contributions

JPM, PG, MGV and JC designed the study, collected the data, analysed and prepared the first draft. All authors put forward different ideas, contributed to the interpretation of the data, early drafts and agreed the final draft.
